# Resonant and non-resonant optimizations by multi-constraint quantum control theory in molecular rotational states

**DOI:** 10.1038/s41598-022-23762-0

**Published:** 2022-11-10

**Authors:** Jin-Fang Li, Jie-Ru Hu, Qiu-Fen Guo, Dong-Shan He

**Affiliations:** 1 Department of Physics and Electronic Engineering, Xianyang Normal University, Shaanxi, 712000 China; 2 State Key Laboratory of Precision Spectroscopy, Department of Physics, East China Normal University, Shanghai, 200062 China

**Keywords:** Information theory and computation, Quantum physics

## Abstract

It is a promising research for optimization of quantum gate in the field of quantum computation. We investigate the feasibility of implementing the single-qubit gate (Hadamard) in molecular rotational system. By applying the Multi-constraint quantum optimal control method, the excepted final states can be achieved based on the molecular rotational states both in resonant and non-resonant cases with the control pulses. The permanent electric dipole moment is ignored in non-resonance. Besides, the zero-pulse area constraint and the constant fluence constraint are employed to optimize shapes of control pulses. Finally, we show that the Hadamard gate can be realized with the high fidelity (0.9999) and also examine the dependence of the fidelity on pulse fluence as well as the control pulse.

## Introduction

The important process of quantum computation is the control of quantum logical gates^1–4^. In 2002, DeMille proposed physical realization of a quantum computer based on an array of trapped polar diatomic molecules^5^. Then, more and more protocols about molecular quantum states have been suggested as physical systems for quantum computation^6–18^. The structure of molecule is multifarious, and schemes have been proposed for implementing quantum bits with laser pulses to operate quantum logic gates in different molecular states, such as, vibrational states^6–8^, rotational states^8–10^, and even hyperfine levels of electronic ground states^11–13^. Furthermore, quantum logical gates can also be optimized in the molecular pendular states which has been studied specifically in Refs.^15–17^. Recently, the high fidelities of entanglement have been demonstrated between the rotational states of a molecular ion and the internal states of an atomic ion^19^.

A general quantum gate can always be decomposed into one- or two- qubit unitary transformation^1,20^, and quantum gates stored quantum information can be controlled by the coherent laser pulse^21,22^. Recently, a machine learning for quantum state transformations was proposed with high accuracy^23^ and a CNOT gate was generated when the parameters were set reasonably between two atoms^24^.

Two-level quantum system has its ubiquitous presence in physics, such as in the basic ideas of bonding of atoms based on the superposition of two quantum states, in quantum optics and laser physics, in magnetic resonance and a multitude of other phenomena^25^. The qubits $|0\rangle$ and $|1\rangle$ are always considered as quantum basis corresponding to two eigenstates in two-level system^26–30^. The system with close energy and large energy difference from other energy levels is approximately considered as actual two-level system, and the influence of external interference on this system is ignored.

The above researches were executed with resonant pulses based on the coupling between an electric dipole moment and the control pulse. Actually, in quantum thermodynamics, the non-resonant two-level transitions have been analyzed where the mode frequency is no need to match the transition frequency^31,32^. The authors demonstrated under the assumption that the design parameters generated during the algorithm are non-resonant, some physical problems are efficiently solvable by gradient descent^33^. During the non-resonant optimization^34–36^, the permanent dipole moment can be ignored while the induced dipole moment (due to the polarization of electrons) plays an important role. When the molecular system is subjected to an intense non-resonant laser pulse^37^, then the axis of molecules aligns with the polarization direction of the field, which have been demonstrated experimentally^38^. With the non-resonant control pulse, the optimization for quantum states were studied based on vibrational^36^ and rotational^39^ states of molecules respectively.

The quantum optimal control method for optimization of unitary operation is proposed in Ref.^40^, the crucial step of this method is to optimize quantum operator based on Schrödinger equation. Finally, the unitary operator is applied in any initial state to optimize quantum logical gate to the final state. This theory is applied extensively in schemes^40–46^, Shu and collaborators promote this theory by adding two constraints named zero-area and constant-fluence on control pulse^42,43,45,46^.

The purpose of this work is to investigate the optimization of Hadamard gate with resonant pulse, and simultaneously, the possibility of implementing Hadamard quantum gate based on non-resonant interaction is also studied which is actually the Raman-type process. Quantum optimization for quantum gates can be accomplished with control pulse by different methods, such as the Strongly Modulating Pulses^47,48^, the Gradient Ascent method^49^ and the adapted strongly modulating pulse^50^. Here, the quantum optimal control method mentioned in the above paragraph is applied both in the resonant and non-resonant excitation cases in this work. In addition, in this method the two important constraints (i.e., no-dc component and constant-fluence) are imposed on the pules, so that the time-dependent control pulse is keeping the energy of pulse unchanged as compared with the initial condition. Specifically, we employ this method to realize Hadamard gate based on rotational states of the CO molecular system, and the high fidelity can be achieved with the ideal population for evolution of the quantum states. Besides, the phase evolution of quantum states is also discussed. Even though the optimization with high fidelity is a challenge for quantum computation with an admissible error lower than $10^{-4}$, but our results show that the high fidelity (0.9999) for optimization of Hadamard quantum gate can be achieved both in resonant and non-resonant cases. The advantage for non-resonant case is that the carrier frequency of the control pulse which is not required to correspond to energy difference which will be helpful for theoretical work to be carried out experimentally.

The remainder of this article is organized as follows. In “Method” section, the quantum optimal control method is described specifically. Then we analyze quantum gate with numerical simulation based on molecular system in “Simulation” section, and the main results are summarized in “Discussion” section.

## Method

In this section, we introduce the quantum optimal control theory named Multi-constraint optimal control theory. The time evolution of the wave function from the initial state $\Psi (0)$ can be described by $\Psi (t)={U}(t,0)\Psi (0)$ with *U*(*t*, 0) the corresponding unitary evolution matrix, which is governed by time-dependent Schrödinger equation^42,45^

1 $$\begin{aligned} i\dfrac{\partial \Psi (t)}{\partial t}=\hat{H}(t) \Psi (t), {U}(0,0)=\mathbb {I}, \end{aligned}$$

with $\hslash =1$ (atomic units are used in this work). The goal of this work is to generate a specified logic gate *H* under the control field *E*(*t*). $U_{T}$ means the unitary matrix at the final moment.

A convenient mathematical formulation of this control objective named as fidelity (F) which can be calculated as

2 $$\begin{aligned} F(U_{T})=\dfrac{1}{2^{n}} \left| \textrm{Tr} \left( H^{\dagger }U_{T} \right) \right| ^{2}= \dfrac{1}{2^{n}} \langle H \vert U_{T}\rangle \langle U_{T}\vert H \rangle , \end{aligned}$$

where $\langle H \vert U_{T} \rangle =\textrm{Tr}(H^{\dagger }U_{T})$, $F(U_{T}) \in [0,1]$ and *n* is the number of qubits.

To optimize the control field for maximizing the objective, a dummy variable $s\geqslant 0$ as used in Ref.^45^ is employed to parameterize the optimization, which can be expressed as

3 $$\begin{aligned} g_{0}(s)\equiv \dfrac{dF(s,U_{T})}{ds}=\int ^{T}_{0} \dfrac{\delta F(s,U_{T})}{\delta E(s,t)} \dfrac{\partial E(s,t)}{\partial s} dt\geqslant 0, \end{aligned}$$

that can be satisfied by updating the control pulse as

4 $$\begin{aligned} \dfrac{\partial E(s,t)}{ \partial s} = \dfrac{\delta F(s,U_{T})}{\delta E(s,t)}. \end{aligned}$$

In the resonant optical control case^41^, the interaction between the dipole-dipole molecule and control field plays an important role, the fidelity can be written as

5 $$\begin{aligned} \dfrac{\delta F_r}{\delta E(t)}=\dfrac{1}{2^{n}} 2 \Re \left( \langle H \vert -i\mu (t)\rangle \langle U_{T}\vert H \rangle \right) . \end{aligned}$$

where ${\mu }(t)= U^{+}(t,0) \mu U(t,0)$, $F_r$ represents the objective in resonance.

Similar to the resonant optical control case^41,42^, the gradient of $F(s,U_{T})$ with respect to the temporal control pulse *E*(*s*, *t*) for the non-resonant control scheme can be derived as (see also Ref.^40^)

6 $$\begin{aligned} \dfrac{\delta F_n}{\delta E(t)}=\dfrac{1}{2^{n}} 2 \Re \left( \langle H \vert -2iU_{T}{\alpha }(t)E(t) \rangle \langle U_{T}\vert H \rangle \right) , \end{aligned}$$

where ${\alpha }(t)E(t)$ with ${\alpha }(t)= U^{\dagger }(t,0) {\alpha } U(t,0)$ is the time-dependent induced-dipole-moment operator and ${\alpha }$ denotes the polarizability, $F_n$ represents the objective function in non-resonance.

7 $$\begin{aligned} \alpha =(\alpha _{\Vert }-\alpha _{\bot }) \left( \cos ^{2}\theta _{1}+ \cos ^{2}\theta _{2} \right) +\alpha _{\bot }. \end{aligned}$$

For practical applications, Eq. (4) may be generalized to include a set of equality constraints $F(E(\cdot ,s))$, on the optimal optical fields

8 $$\begin{aligned} g_{\ell }(s)\equiv \frac{dF_{\ell }(E(\cdot ,s))}{ds}=\int _{0}^{T} \frac{\delta F(E(\cdot ,s))}{\delta E(s,t)}\frac{\partial E(s,t)}{\partial s}dt=0. \end{aligned}$$

The combined demands in Eqs. (3) and (8) can be fulfilled simultaneously by updating the control field as function of variable *s*^42,43^,

9 $$\begin{aligned} \dfrac{\partial E(s,t)}{ \partial s} =S(t) \left\{ g_{0}(s)\sum _{\ell =0}^{M} \left[ \Gamma ^{-1} \right] _{0\ell }PC_{l}(s,t) \right\} , \end{aligned}$$

The parameters $c_{0}(s,t)$ and $c_{l}(s,t)$ can be described as

10 $$\begin{aligned} c_{0}(s,t)= & {} \frac{\delta F_{0}(E(s,t))}{\delta E(s,t)}, \end{aligned}$$

11 $$\begin{aligned} c_{l}(s,t)= & {} \frac{\delta F_{\ell }(E(s,t))}{\delta E(s,t)}, (\ell =1,2,\ldots , M), \end{aligned}$$

$\Gamma$ is an invertible full rank $(M+1)\times (M+1)$ square matrix composed of elements

12 $$\begin{aligned} \Gamma _{\ell +1,\ell '+1}=\int _{0}^{T}S(t)c_{l}(s,t)c_{l'}(s,t)dt. \end{aligned}$$

The project vector P can act on an arbitrary function $\alpha (s,t)$

13 $$\begin{aligned} P\alpha (s,t)\equiv \int _{0}^{T}S(t')\left( \sum _{k,k'=0}^{M}c_{k}(s,t) \left[ \Gamma ^{-1} \right] _{kk'}c_{k'}(s,t') \right) \alpha (s,t')dt', \end{aligned}$$

And then we take (9) into (3) and (8),

$$\begin{aligned} g_{l'}= & {} g_{0}(s)\int _{0}^{T}S(t)c_{l'}(s,t)\sum _{l=0}^{M} \left[ \Gamma ^{-1} \right] _{0l}Pc_{l}(s,t)dt\\= & {} g_{0}(s)\delta _{0l'} \end{aligned}$$

where $\ell '=0,1,2,\ldots , M$; then the control field as function of variable *s*

14 $$\begin{aligned} \dfrac{\partial E(s,t)}{ \partial s} =S(t)\sum _{\ell =0}^{M} \left[ \Gamma ^{-1} \right] _{0\ell }c_{l}(s,t), \end{aligned}$$

where $S(t)\ge 0$ is an envelope function which smoothly turns on and off the control field. Here the optimized control field is limited to satisfy two constraints simultaneously

15 $$\begin{aligned} J_1\equiv \int ^{T}_{0} E(s,t)dt = 0, \end{aligned}$$

and

16 $$\begin{aligned} J_2\equiv \int ^{T}_{0} E^{2}(s,t)dt = \mathcal {C}. \end{aligned}$$

The zero-pulse area constraint in Eq. (15) implies that the optimized control field does not contain dc-components, leading to pure ac control, whereas the constant fluence constraint in Eq. (16) keeps the energy of the optimized fields unchanged as compared with the initial guess. The numerical details of performing this multiple constraint quantum optimal control method can be found in Refs.^42,43^.

## Simulation

### Optimization for Hadamard in resonance

In the case of dipole-dipole approximation, the Hamiltonian of control field acting on the molecular system can be written as^51–53^

17 $$\begin{aligned} H(t)=B{{\textbf {J}}}^{2}-\mu E(t)\cos \theta , \end{aligned}$$

where *B* is rotational constant, $\mu$ is inherent dipole moment, ${{\textbf {J}}}$ is the total rotational angular momentum and $\theta$ is the degree between the molecule axis and control field. According to the Ref.^5^, DeMille proposed that two quantum states can be described two energy levels of diatomic molecule. Then we choose two lowest energy levels as the orthogonal complete quantum basis, which can be described by^15^

18 $$\begin{aligned} \langle \theta ,\varphi \vert V_{0} \rangle =Y_{0,0}(\theta ,\varphi ); \langle \theta ,\varphi \vert V_{1} \rangle =Y_{1,0}(\theta ,\varphi ). \end{aligned}$$

Then the control pulse is added to this rotational system and the input form of pulse is assumed as: $E(t)=S(t)E_{0}\cos (\omega _{0} t)$, where *S*(*t*) is envelope function: $S(t)=$ exp $[-4$ ln $2(t-t_{0})^2/\tau ^2]$, its FWHM is $\tau =4.5\times 10^{5}$ a.u. (the atomic units are used), $t_{0}=T/2=9\times 10^{5}$ a.u., *T* is total time; $E_{0}=1\times 10^{-4}$ a.u. is amplitude. During the numerical simulation, we set the numbers of steps $N=2\times 10^{4}$, and then the time discretization $t=90$ (a.u.) based on the above total time T. The dummy variable *s* in Eq. (3) is set as $2\times 10^{-5}$ (unit less). $\omega _{0}$ equals to the difference of two-level energy. The initial pulse with Gaussian envelope can control the initial states to the final states after enough iteration numbers. As a consequence, the optimized pulse that we need is the one corresponding to the last iteration.

In Table 1, we give the details for Hadamard with different initial states and the initial phase $\varphi$ is set as $\pi /6$.

**Table 1:** The simulation results of Hadamard for different initial states.

	Initial state	Final state	$F_{r}$/$F_{n}$
(i)	$\vert 0 \rangle e^{-i\varphi }$	$\dfrac{1}{\sqrt{2}} (\vert 0 \rangle e^{-i\varphi _{1}} + \vert 1 \rangle e^{-i\varphi _{2}} )$	0.9999
(ii)	$\vert 1 \rangle e^{-i\varphi }$	$\dfrac{1}{\sqrt{2}} (\vert 0 \rangle e^{-i\varphi _{1}} - \vert 1 \rangle e^{-i\varphi _{2}} )$	0.9999
	$(sin\dfrac{\pi }{4} \vert 0 \rangle + cos\dfrac{\pi }{4} \vert 1 \rangle )e^{-i\varphi }$	$\vert 0 \rangle e^{-i\varphi _{1}}$	
(iii)	$(sin\dfrac{\pi }{4} \vert 0 \rangle - cos\dfrac{\pi }{4} \vert 1 \rangle )e^{-i\varphi }$	$\vert 1 \rangle e^{-i\varphi _{2}}$	0.9999

**Figure 1:**
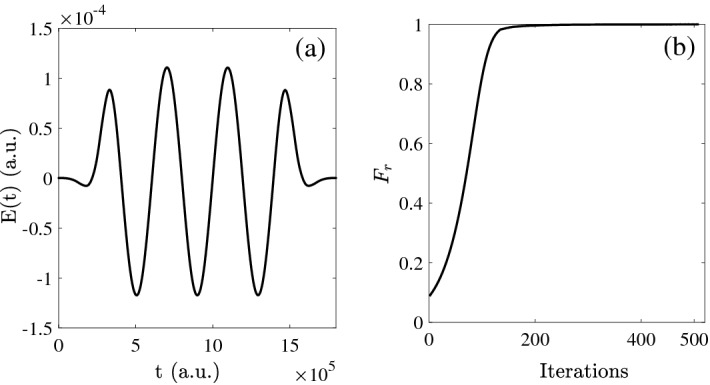
(**a**) The behavior of control field as the function of time; (**b**) The fidelity as the function of iterations.

Under the control field, based on the rotational energy level of CO molecule ($\mu =0.044$ a.u.), we will optimize the Hadamard logical gate. The condition for the resonance is that the difference of energy equals to the frequency of field. In Fig. 1a, we give the evolution of control filed as function of time. From Fig. 1b we can find that the fidelity can reach 0.9999 after 507 iterations. Meanwhile, the control pulse is output in the last iteration.

**Figure 2:**
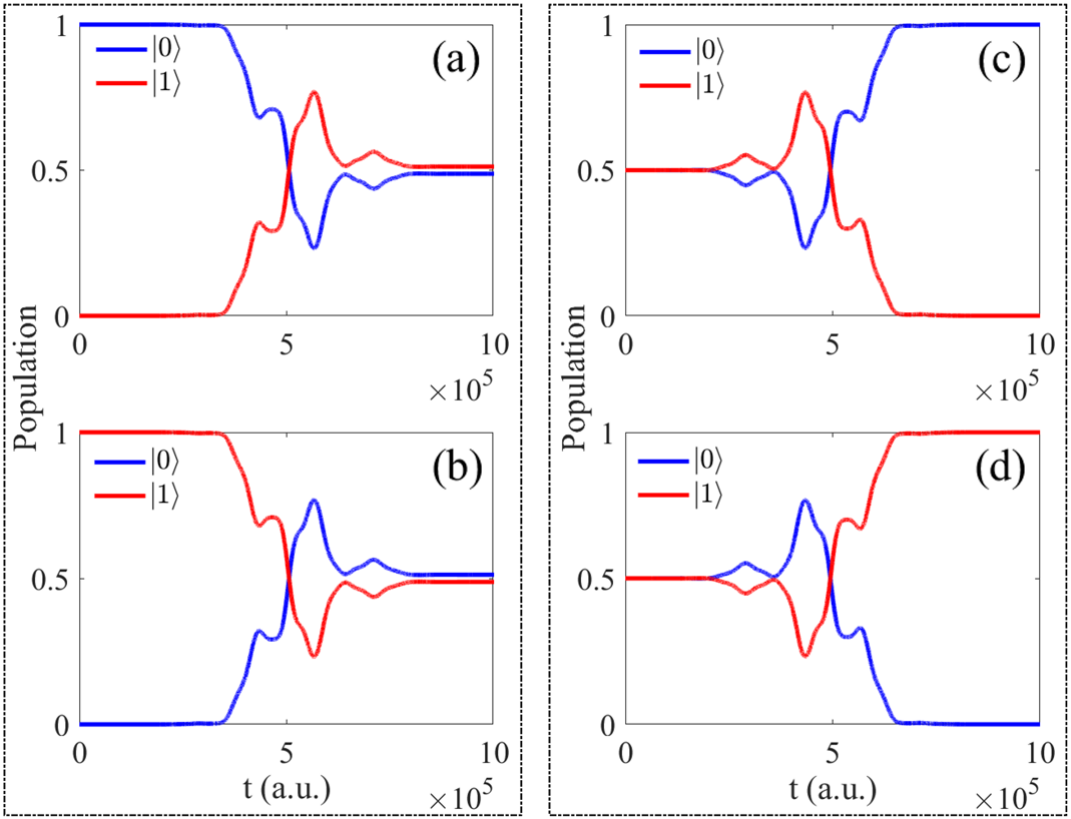
The population as function of time for different initial states: (**a**) $\vert 0 \rangle e^{-i\varphi }$; (**b**) $\vert 1 \rangle e^{-i\varphi }$; (**c**) $\sqrt{1/2}(\vert 0 \rangle +\vert 1 \rangle )e^{-i\varphi }$; (**d**) $\sqrt{1/2}(\vert 0 \rangle -\vert 1 \rangle )e^{-i\varphi }$.

The initial states and the final states are given in theory and the corresponding population behavior are given in Fig. 2. By the analysis, when the initial state is $\vert 0 \rangle e^{-i\varphi }$, the population of final state are 0.4878 for $\vert 0 \rangle$ and 0.5122 for $\vert 1 \rangle$. The initial state is $\vert 1 \rangle e^{-i\varphi }$, the population of final state are $\vert 0 \rangle$ for 0.5122 and $\vert 1 \rangle$ for 0.4878, the population of final states are reversed. Both of the above initial states, the population of final states should be 0.5000 in theory after operating by Hadamard gate. When the initial state is $\sqrt{1/2}(\vert 0 \rangle +\vert 1 \rangle )e^{-i\varphi }$, the population of final state are $\vert 0 \rangle$ for 0.9998 and $\vert 1 \rangle$ for $1.7206\times 10^{-4}$, the difference of population is around $10^{-4}$. In addition, when the initial state is $\sqrt{1/2}(\vert 0 \rangle -\vert 1 \rangle )e^{-i\varphi }$, then the final population are $\vert 1 \rangle$ for 0.9998 and $\vert 0 \rangle$ for $1.7206\times 10^{-4}$, the population of final states are also reversed, and the final population should be 1 and 0 exactly in theory.

**Figure 3:**
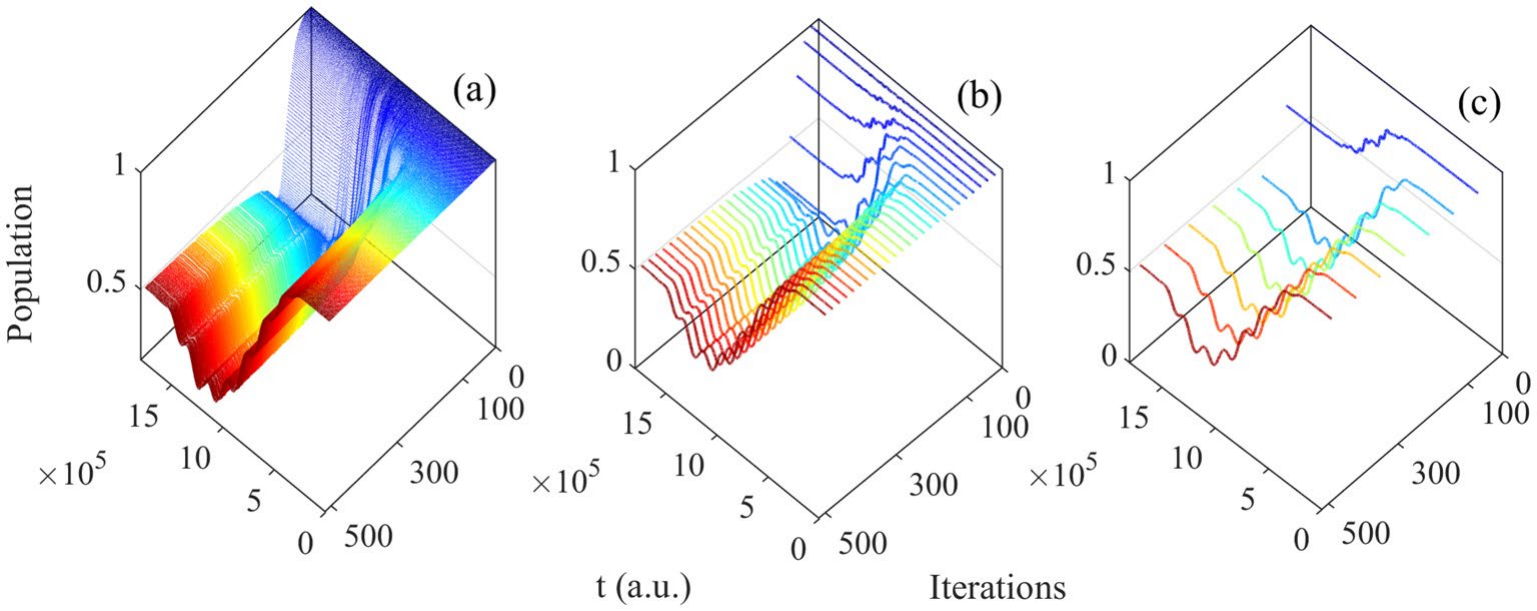
The population of basis $\vert 0 \rangle$ as function of time and iterations: (**a**) all iterations; (**b**) every 20 times; (**c**) every 70 times.

**Figure 4:**
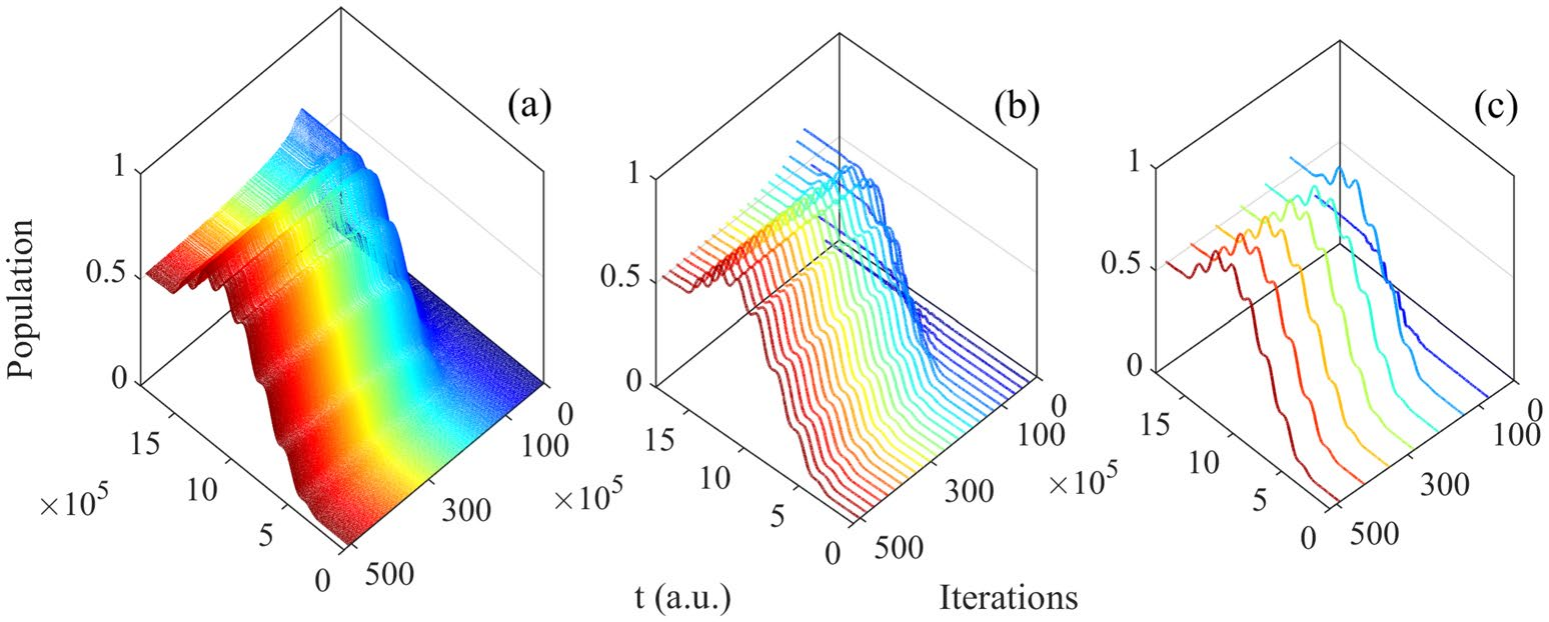
The population of basis $\vert 1 \rangle$ as function of time and iterations: (**a**) all iterations; (**b**) every 20 times; (**c**) every 70 times.

Following, we choose the initial state $\vert 0 \rangle e^{-i\varphi }$ (Fig. 2a) as example in this section and analyze the basis of $\vert 0 \rangle$ and $\vert 1 \rangle$. The population can be seen as the function of time and iterations, which is shown in Figs. 3, 4 respectively.

From Figs. 3, 4, it can be seen the evolution of population based on both time and iterations. It can be seen more clearly from above two figures by taking every 20 and 70 iteration times, the evolution of two basis are reversal which is satisfied with the operation of Hadamard gate.

**Figure 5:**
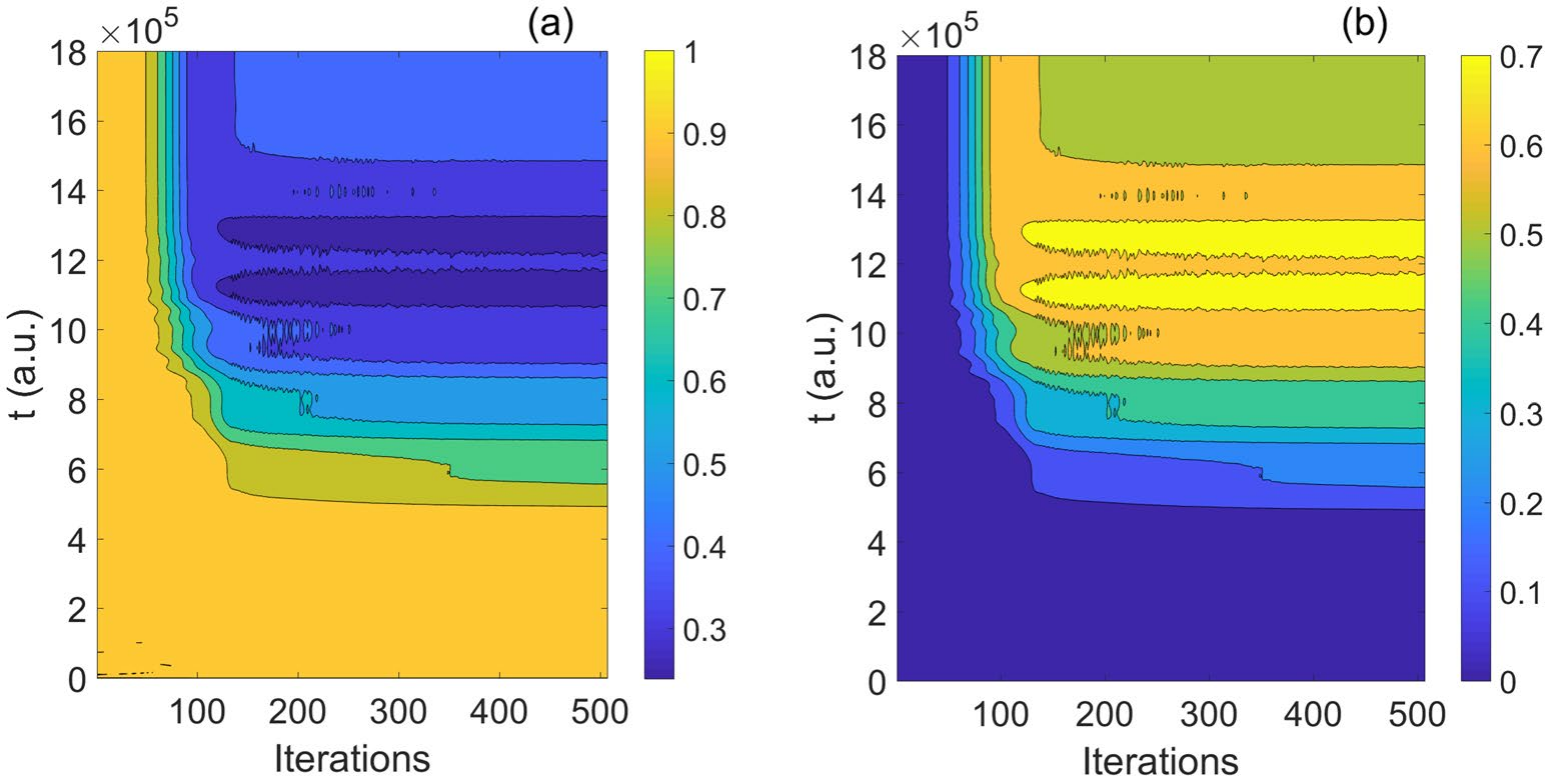
(Contour) The evolution of population as function of iterations and time; (**a**) the evolution of $\vert 0 \rangle$; (**b**) the evolution of $\vert 1 \rangle$.

Here, we give the results of population $\vert 0 \rangle e^{-i\varphi }$ and $\vert 1 \rangle e^{-i\varphi }$ in Fig. 5a,b. With increasing of the iterations and time, the final population are coincided with theoretical results of Hadamard gate. From the variation of color, it can be seen the evolution is complex and rapid at the beginning, but with the increasing time and after 120 iterations, the difference with color becomes small, which means the change amount of objective decrease. So the results anastomose with phenomenon shown in Fig. 2a.

### Constraints and without constraints

Based on the theory shown in “Method” section, by adding the constraints (Eqs. 15, 16) on initial field can help the control pulse keep the zero-area and constant-fluence^43,45^. Here, it is necessary to discuss optimize the specific quantum logical gate (Hadamard gate in this work) to analyze the difference with and without constraints. During the above resonant optimization, we get the results with constraints and the optimized control pulse in Fig. 1a.

**Figure 6:**
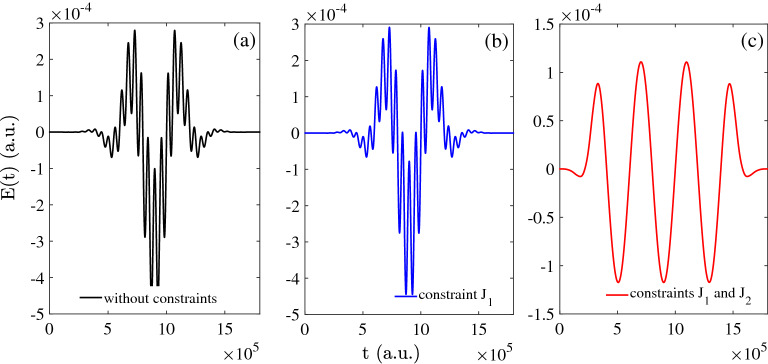
The control field as function of time: (**a**) without constraints; (**b**) with the constraint $J_{1}$; (**c**) with the constraints $J_{1}$ and $J_{2}$.

In Fig. 6, the control pulse are plotted as function of evolution time for the final iteration under different conditions. In Fig. 6a, we give the optimized control pulse when the constraints have not added on the initial pulse. Then in Fig. 6b, we keep the constraint $J_{1}$ (Eq. 15 on initial pulse which means only keep the condition of control field with zero-area. In addition, in Fig. 6c, the constraints Eqs. 15, 16) are both kept on the initial pulse so the fluence of control field is constant, and the amplitude of the optimized field is around $10^{-4}$ a.u.

**Figure 7:**
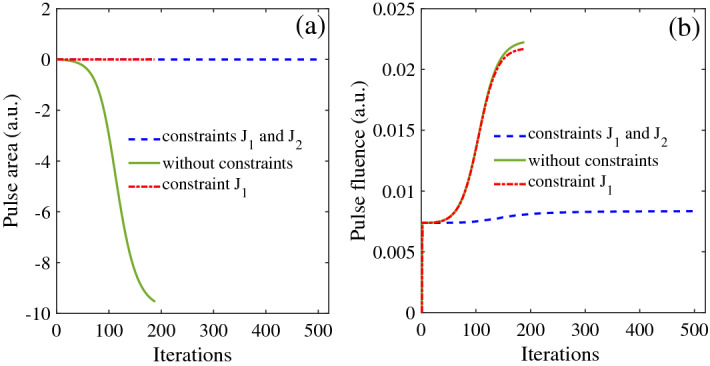
The green line without constraints; The red line with constraint $J_{1}$; The blue line with constraints $J_{1}$ and $J_{2}$: (**a**) the area of of control field; (**b**) the fluence of control field.

In Fig. 7, the area and fluence of the optimized control pulse are discussed numerically. In Fig. 7a, the area are kept zero with both one constraint and two constraints. Meanwhile, in Fig. 7b, the fluence of control pulse are kept constant with the increasing of iterations under two constraints and the constant value is 0.0055 a.u.. It is found that the fluence of control pulse increase monotonously which is not useful to optimize the pulse.

After the final iteration, the control pulse are expected smoothly with two constraints. The theoretical results for control pulse presented in this work is useful to general optimization for quantum logical gates subject to an arbitrary number of equality constraints in the time domain. Considering the constraints, based on the above calculation, the results are achieved with the constrains. Then, we will discuss the optimization of Hadamard gate in non-resonance in same physical system with two basis $\vert 0 \rangle$ and $\vert 1 \rangle$ and initial states. In following section, the optimization of Hadamard gate in non-resonance are based on the theory with constraints due to the above advantage.

### Optimization for Hadamard in non-resonance

In this section, we will analyze the Raman process^54,55^, the interaction is between the control pulse and polarization of molecule during this process. By applying the condition of polarization, the Hamiltonian can be written as^54^

19 $$\begin{aligned} H(t)=B{{\textbf {J}}}^{2}-\dfrac{1}{2} E^{2}(t)[(\alpha _{\Vert }-\alpha _{\bot })\cos ^{2}\theta +\alpha _{\bot }], \end{aligned}$$

$\alpha _{\Vert }$ and $\alpha _{\bot }$ are parallel and vertical polarizability component. However, the inherent dipole moment of molecule plays an important role in the two-level system while the parallel and vertical polarizability component are crucial in the non-resonance case^36^. That means the inherent dipole moment is ignored in non-resonance.

According to the Selection Rule^54^, we choose the $\vert \tilde{J}_{0}\rangle$ and $\vert \tilde{J}_{2}\rangle$ corresponding to two-level states in rotational molecular system, the basis consisted of spherical harmonic functions are expended as

20 $$\begin{aligned} \langle \theta ,\varphi \vert V_{0} \rangle =Y_{0,0}(\theta ,\varphi ); \langle \theta ,\varphi \vert V_{2} \rangle =Y_{2,0}(\theta ,\varphi ). \end{aligned}$$

**Figure 8:**
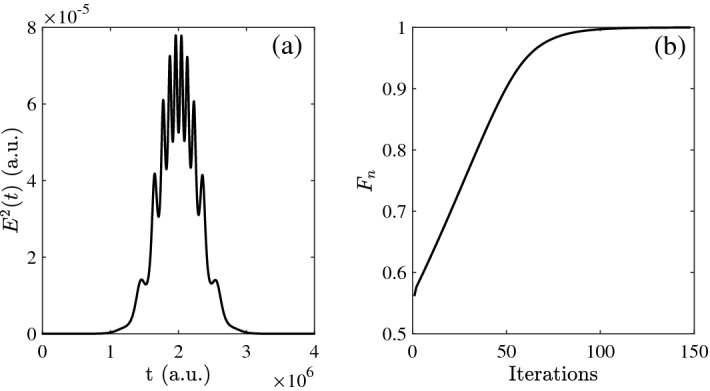
(**a**) The evolution of the square of control pulse as function of time; (**b**) The evolution of fidelity as function of the iterations.

Then the control field is applied in the rotational states of CO molecule to optimize the Hadamard gate. Assuming the initial field is $E(t)=S(t)E_{0}\cos (\omega _{0} t)$, and *S*(*t*) is envelope function which is similar to the resonant case: $S(t)=$exp$[-4$ln$2(t-t_{0})^2/\tau ^2]$, and the FWHM is $\tau =1\times 10^{6}$ a.u., $t_{0}=T/2=2\times 10^{6}$ a.u., *T* is total time. Actually, for this case, the evolution time is little longer than in resonance after our numerical simulation. The square amplitude of pulse is $E_{0}^{2}=8\times 10^{-5}$ a.u.. The numbers of steps $N=2\times 10^{3}$, and then the time discretization $t=2\times 10^{3}$ (a.u.) based on the above total time T. The dummy variable *s* in Eq.(8) is set as $9\times 10^{-5}$ (unit less). Besides, $\omega _{0}$ (the central frequency) should be lager than the difference of two energy levels. Here, we take the population of four different initial states with the same initial states in resonance. And corresponding comparison for optimized fidelity is shown in Table 1. Then the optimized pulse is plotted as function of iterations in Fig. 8a, the peak value is around 8$\times 10^{-5}$ a.u.. From the Fig. 8b, it can be seen when the iterations reach 147, then the fidelity can approach 0.9999. The numbers of iteration are much less the resonant results in Fig. 1b.

**Figure 9:**
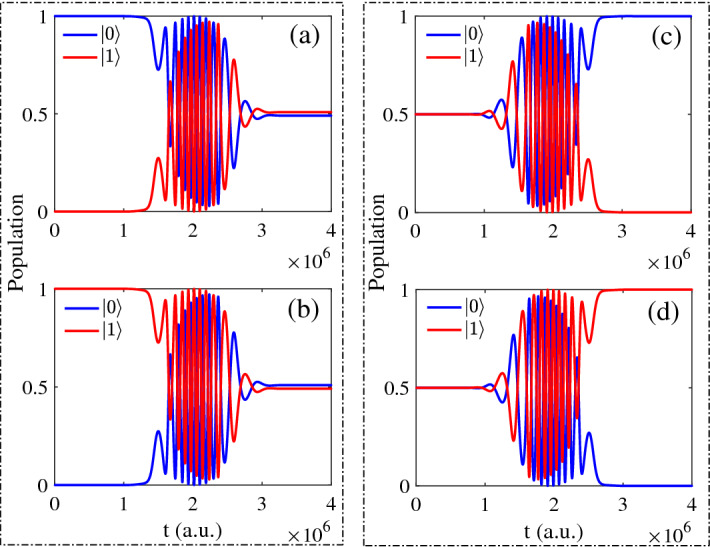
In non-resonant case for different initial states: (**a**) $\vert 0 \rangle e^{-i\varphi }$; (**b**) $\vert 1 \rangle e^{-i\varphi }$ (**c**) $\sqrt{1/2}(\vert 0 \rangle +\vert 1 \rangle )e^{-i\varphi }$ (**d**) $\sqrt{1/2}(\vert 0 \rangle -\vert 1 \rangle )e^{-i\varphi }$.

In Fig. 9, the phenomenon of population is similar to the resonant case in Fig. 2, but the oscillation is much severely. By the analysis, when the initial state is $\vert 0 \rangle e^{-i\varphi }$, the population of final state are 0.4912 for $\vert 0 \rangle$ and 0.5088 for $\vert 1 \rangle$. The initial state is $\vert 1 \rangle e^{-i\varphi }$, the population of final state are $\vert 0 \rangle$ for 0.5088 and $\vert 1 \rangle$ for 0.4912, the population of final states are reversed. When the initial state is $\sqrt{1/2}(\vert 0 \rangle +\vert 1 \rangle )e^{-i\varphi }$, the final state is $\vert 0 \rangle$ for 0.9999 and $\vert 1 \rangle$ for 0.0001, the difference of population is around $10^{-4}$. In addition, when the initial state is $\sqrt{1/2}(\vert 0 \rangle -\vert 1 \rangle )e^{-i\varphi }$, then the population of final states are $\vert 1 \rangle$ for 0.9999 and $\vert 0 \rangle$ for 0.0001. Compared to the results in resonance, these population of final states are more closer to the theoretical values to satisfy Hadamard gate.

**Figure 10:**
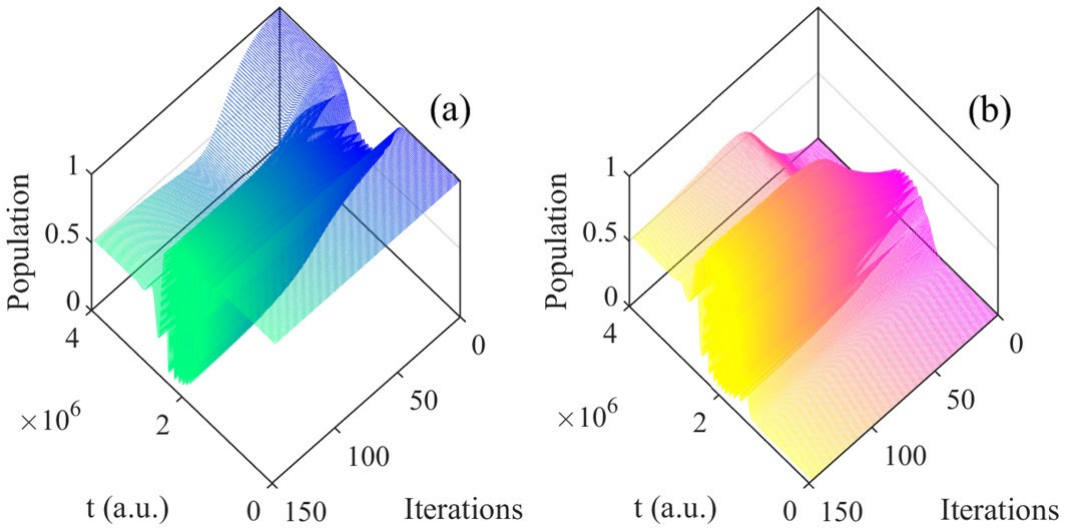
The population plotted as function of time and iterations: (**a**) the basis $\vert 0 \rangle$; (**b**) the basis $\vert 1 \rangle$.

**Figure 11:**
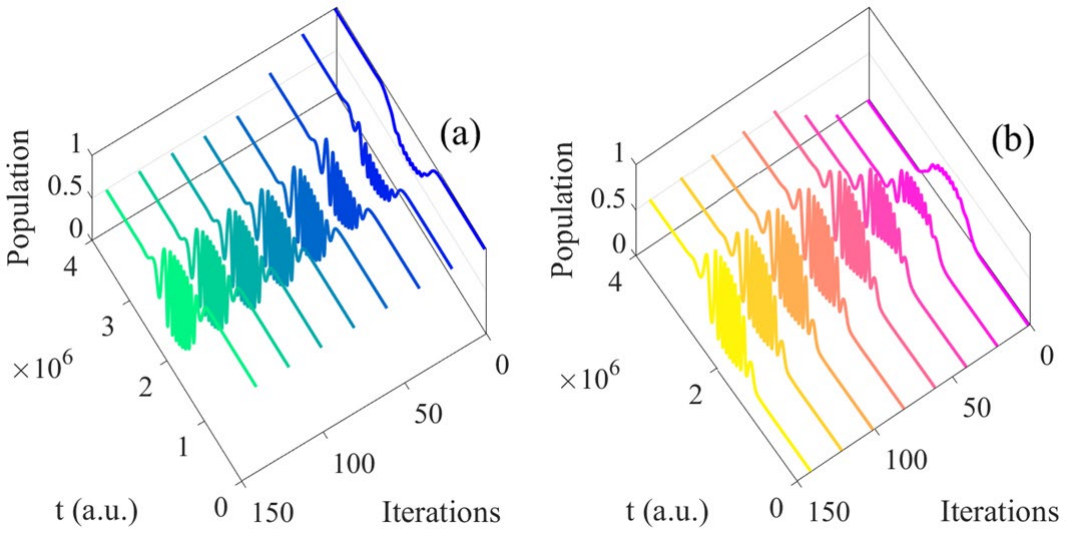
The population plotted as function of time and iterations (every 20 times): (**a**) the basis $\vert 0 \rangle$; (**b**) the basis $\vert 1 \rangle$.

**Figure 12:**
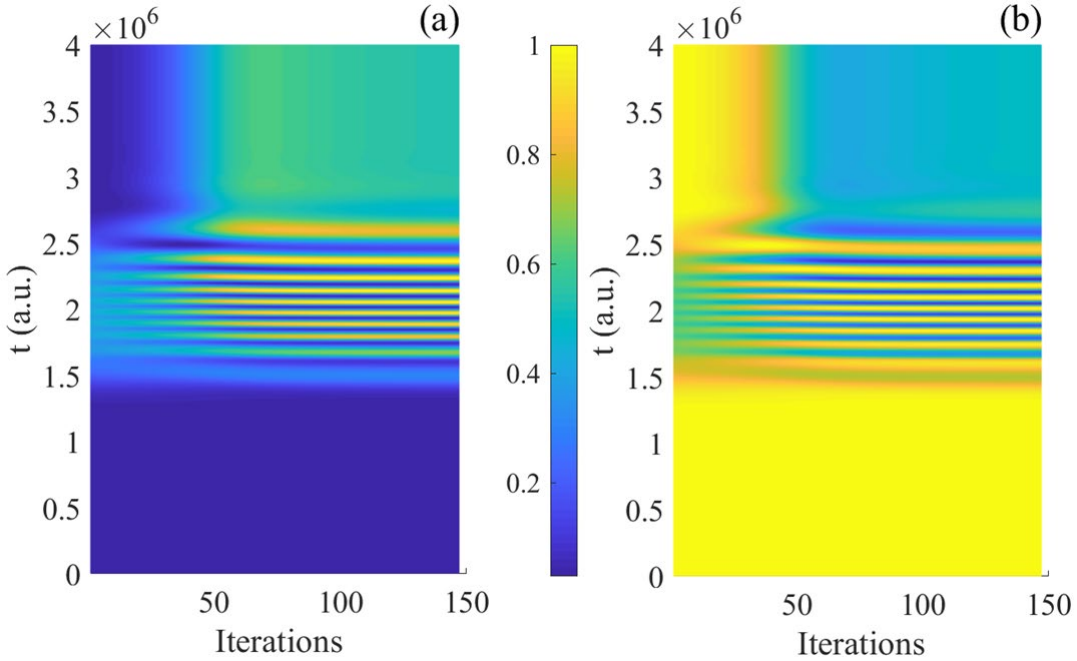
(Contour) The population of quantum basis as function of time and iterations: (**a**) the basis $\vert 1 \rangle$; (**b**) the basis $\vert 0 \rangle$.

Then the initial state $\vert 0 \rangle e^{-i\varphi }$ (Fig. 9a) is analyzed which is similar to the one in resonance, and the evolution of basis are shown in Fig. 10. With the closer evolution time and same initial states, the numbers of iteration are less than in resonance. Meanwhile, the evolution of population are both satisfied operation of Hadamard gate. Besides, in order to clarify the specific detail, we give the evolution of population as function of time and iterations every 20 times in Fig. 11. In addition, it can be seen the population is much intensive in middle of evolution in Fig. 12 compared to the resonant results in Fig. 5, and with the increasing time and after around 50 iterations, the change amount of fidelity decrease. Based on the above analysis, it can be found that the control pulse can keep zero-area and constant-fluence with constraints both in resonance and non-resonance, and fidelity can reach 0.9999. The shape of control pulse keep smooth which can be helpful to improve the possibility of realizing pulse in experiments.

### Phase analysis

The evolution of phase can carry the information of quantum state in the optimization process for quantum state. We hope that the optimization of quantum logic gates can be carried out within the error range so that the evolution phase can be considered as global phase^56–58^.

In Table 2, we also give the phase evolution both in resonant and non-resonant cases (the unit of phase is deg).

**Table 2:** The analysis of phases evolution (the unit of phase is (*deg*)).

$\Delta \varphi =\vert \varphi _{2}-\varphi _{1}\vert$	Resonance	Non-resonance
(i)	0.4924	0.2503
(ii)	0.4885	0.2418
(iii)	0.4841	0.2395

From Table 2, it can be found that the quantum phases of final states are 0.4924, 0.4885 and 0.4841 in resonance and 0.2503, 0.2418 and 0.2395 in non-resonance respectively with the same initial phase ($\pi /6$), it means that the phases of final state are obtained better in non-resonance than in resonance, but both of the results can be seen as global phases within the error range.

## Discussion

In summary, we study the optimization of single-qubit Hadamard gate based on rotational state of molecular two-level dynamic system. The optimization results of the two cases of resonance and non-resonance are obtained respectively. In both cases, we can obtain high fidelity 0.9999 and the population are agreement with the theoretical values. The numbers of iteration in resonance required are less than that of non-resonance, while the pulse intensity and pulse duration required for the resonance are smaller than the non-resonant ones. The important reason is that the lager frequency (energy) difference requires high intensity and much time of control pulse in order to reach the same ideal fidelity. The population of final states and phase in the non-resonant case are better maintained. The evolution phase of quantum states should satisfy the requirements of global phase based on the optimization of the dynamic system and the phase difference is maintained within 1 (deg) in this paper. In addition, we have specifically analyzed that under the given constraints, the obtained pulse can maintain zero-area and constant-fluence, which can be considered as ideal pulse shape, and achieve the purpose of pulse shaping to a certain extent. The results shown in this work about the optimization of quantum logical gates may be useful and feasible for quantum computing involving the molecular quantum states for some kinds of experiment application.

Besides, the research for enhancing the coherence time of the ultracold polar molecules have been studied in experiments. For example, the rotational coherence time of KRb can be extended to tens of milliseconds with the assistance of spin-echo technique^59^. The authors extend the rotational coherence to 8.7(6) ms in a dilute gas of polar $^{23}$Na$^{40}$K in an optical trap^60^. For ultracold dipolar molecules at sub-microkelvin temperatures, the coherence time between nuclear spin states of $^{23}$Na$^{40}$K in the singlet rovibrational ground state has also been extend to second-scale experimentally^61^, which is helpful for applying molecules as a versatile quantum memory and for precision measurements on dipolar quantum matter. In addition, the polar YbF has been cooled to sub-Doppler temperature (lower than 100 $\mu$K) via laser cooling and its coherence time can be exceeded more than 150 milliseconds^62^. Based on the above analysis, we can find that the rotational coherence time for different ultracold polar molecules can reach the scale of milliseconds. In this work, the duration of the optimized laser pulse is around dozens of picoseconds ($1.8\times 10^{6}$ a.u. and $4\times 10^{6}$), which is less than the millisecond coherence time of polar molecules reported in experiments. The related works for implementing the tasks of quantum information processing based on the various states of polar molecules by means of the optimal control have not considered the decoherence effects^9,10,13,16,18^, the main reason is that the rotational states of the ultracold molecules at low temperature have long lifetime, which means that the coherent manipulation and quantum operation based on the polar molecule between different rotational states by control pulses can be completed before the decoherence time. Based on the above analysis, we hope the scheme proposed in this paper can be helpful for realization in experiments.

## Data Availability

The data generated and/or analysed during the current study are not publicly available for legal/ethical reasons but are available from the corresponding author on reasonable request.
